# Potential effects of ultraviolet radiation reduction on tundra nitrous oxide and methane fluxes in maritime Antarctica

**DOI:** 10.1038/s41598-018-21881-1

**Published:** 2018-02-27

**Authors:** Tao Bao, Renbin Zhu, Pei Wang, Wenjuan Ye, Dawei Ma, Hua Xu

**Affiliations:** 1 Anhui Province Key Laboratory of Polar Environment and Global Change, School of Earth and Space Sciences, University of Science and Technology of China, Hefei, 230036 China; 2 State Key Laboratory of Soil and Sustainable Agriculture, Institute of Soil Science, Chinese Academy of Sciences, Nanjing, 210008 China

## Abstract

Stratospheric ozone has begun to recover in Antarctica since the implementation of the Montreal Protocol. However, the effects of ultraviolet (UV) radiation on tundra greenhouse gas fluxes are rarely reported for Polar Regions. In the present study, tundra N_2_O and CH_4_ fluxes were measured under the simulated reduction of UV radiation in maritime Antarctica over the last three-year summers. Significantly enhanced N_2_O and CH_4_ emissions occurred at tundra sites under the simulated reduction of UV radiation. Compared with the ambient normal UV level, a 20% reduction in UV radiation increased tundra emissions by an average of 8 μg N_2_O m^−2^ h^−1^ and 93 μg CH_4_ m^−2^ h^−1^, whereas a 50% reduction in UV radiation increased their emissions by an average of 17 μg N_2_O m^−2^ h^−1^ and 128 μg CH_4_ m^−2^ h^−1^. No statistically significant correlation (P > 0.05) was found between N_2_O and CH_4_ fluxes and soil temperature, soil moisture, total carbon, total nitrogen, NO_3_^−^-N and NH_4_^+^-N contents. Our results confirmed that UV radiation intensity is an important factor affecting tundra N_2_O and CH_4_ fluxes in maritime Antarctica. Exclusion of the effects of reduced UV radiation might underestimate their budgets in Polar Regions with the recovery of stratospheric ozone.

## Introduction

Atmospheric nitrous oxide (N_2_O) and methane (CH_4_) are two main greenhouse gases (GHGs). In addition, N_2_O partly contributes to stratospheric ozone depletion^1^. Increases in N_2_O and CH_4_ emissions and their roles in aggravating global warming, have caused great concern in the past three decades^1^. Currently, the fluxes of N_2_O and CH_4_ and their influencing factors have been extensively investigated from subtropical, tropical, temperate terrestrial ecosystems and boreal tundra in the Northern Hemisphere^2–7^. However, the related studies were conducted very late in the Antarctic terrestrial ecosystem.

Recent studies of N_2_O and CH_4_ fluxes from the Antarctic terrestrial ecosystem mainly concentrated on the McMurdo Dry Valleys of continental Antarctica^8–11^, the Antarctic Peninsula and other islands of maritime Antarctica^12–15^. Microtopography, mineralizing substrate availability, soil temperature, soil moisture and O_2_ availability could affect tundra N_2_O or CH_4_ fluxes^9–12,14–17^. Climate change might affect N_2_O and CH_4_ emissions from the tundra, because some soil parameters, e.g., soil moisture and temperature, are associated with microbial activity and the mineralization of organic carbon and nitrogen in soils^17–19^. In addition, significantly enhanced N_2_O and CH_4_ emissions were found from penguin and seal colonies, which have been identified as “hot spots” for N_2_O and CH_4_ emissions in maritime Antarctica because of the high load of readily available organic carbon and nitrogen through penguin or seal excreta^12,14,16^. Nevertheless, the effects of solar ultraviolet (UV) radiation on N_2_O and CH_4_ fluxes have received little attention in the Antarctic tundra.

Strong UV (UV-A and UV-B) radiation has occurred in Antarctica because of the serious destruction of stratospheric ozone^20^. Enhanced UV radiation resulted in a 75% decrease in the ATP content of the microorganisms in the upper water of the Weddell Sea, Antarctica^21^. Pakulski *et al*.^22^ reported a 57% reduction in marine bacteria around Palmer Station during low total ozone column episodes. A significant correlation has been identified between DNA damage in Antarctic pelagic icefish eggs and UV irradiance^23^. The survival rates of Antarctic krill decrease in response to increased UV radiation^24^. Both UV-A and UV-B are major drivers of the decomposition of vegetation litter in the Antarctic terrestrial ecosystem through the process of photodegradation^25–27^. In addition, they have the potential to affect the structure and function of Antarctic mosses, *Ceratodon purpureus* and *Bryum subrotundifolium*^28^ and to influence indirectly the soil microbial populations and activities^26^. UV radiation is also a key regulator of vegetation morphology and genetic processes and is important in vegetation growth^27,29^. Vegetation growth and soil microbial activities are the main factors influencing plant respiration and N_2_O and CH_4_ emissions from the tundra^28^. Sunlight could greatly affect N_2_O and CH_4_ emissions from tundra ecosystem because of O_2_ release via vegetation photosynthesis^30^. The UV-induced release of carbon from plant litter and soils might contribute to global warming^27^. Therefore, it is important to investigate the effects of UV intensity on tundra N_2_O and CH_4_ fluxes and carbon and nitrogen cycles, in maritime Antarctica.

**Figure 1:**
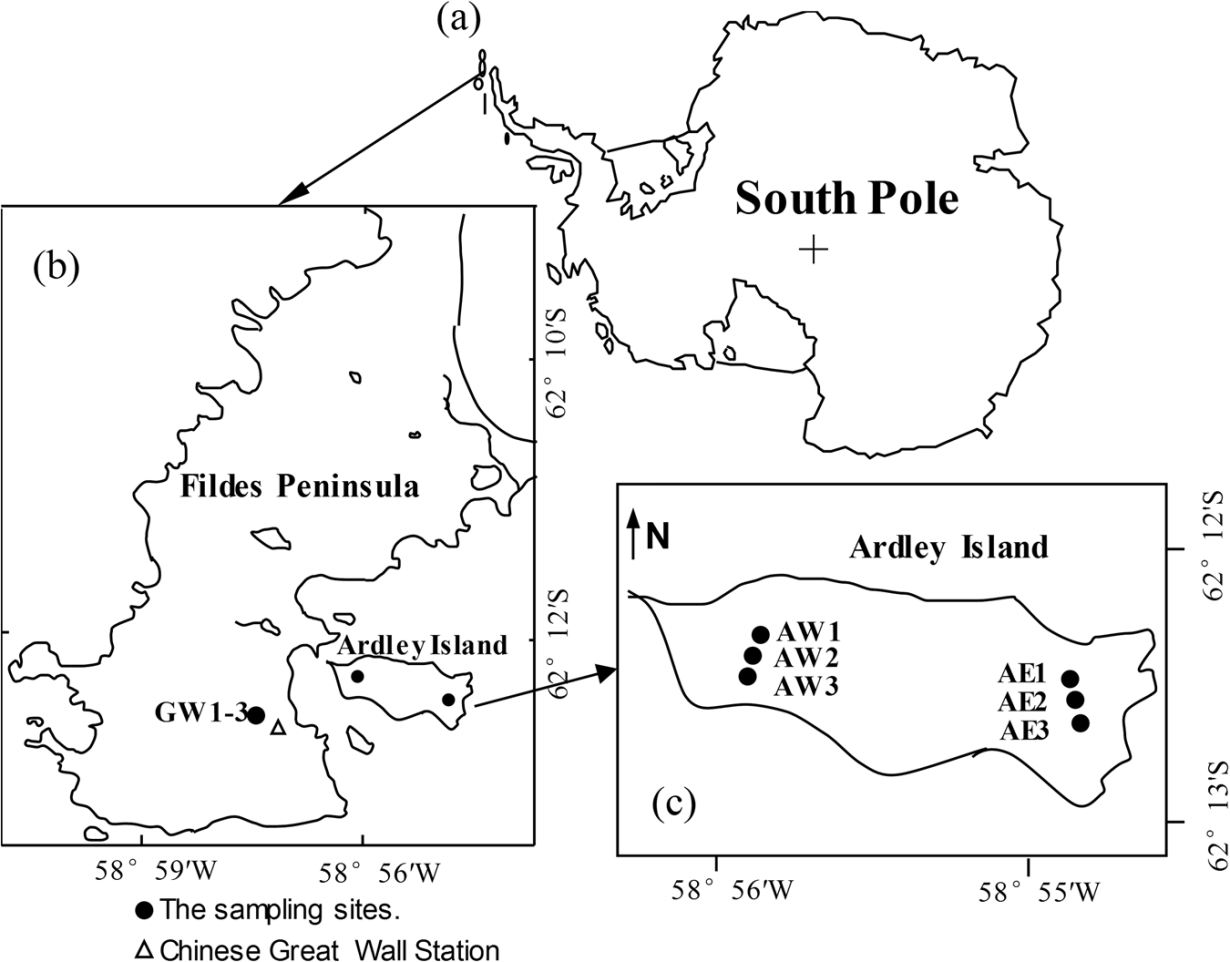
Study area and the N_2_O and CH_4_ fluxes at the observation sites. Panel (a), the dot indicates the location of the investigation area in maritime Antarctica. Panel (b), location of the investigation sites on Fildes Peninsula and Ardley Island, King George Island. Three upland tundra sites (GW1–GW3) are shown. Panel (c), the sites of the flux chambers in the eastern tundra and the western tundra on Ardley Island, including six regular sites AE1–AE3 and AW1–AW3. Note: The map was drawn using CorelDRAW 2017 software (http://www.corel.com/cn/).

Currently, stratospheric ozone has recovered somewhat in Antarctica since the implementation of the Montreal Protocol in 1989^31^. The Antarctic ozone hole has shrunk by nearly 400,000 square miles since it was discovered around 30 years ago. The ozone layer in the Polar Regions is projected to recover to pre-1980 levels by 2048, thus less solar UV radiation will reach the earth’s surface^32^. However, the effects of a reduction in UV radiation on N_2_O and CH_4_ emissions to date have not been investigated in the Antarctic tundra. During the austral summers of 2011/2012, 2013/2014 and 2014/2015, we selected a tundra ecosystem in the maritime Antarctica as study area (Fig. 1) and for the first time, investigated tundra N_2_O and CH_4_ fluxes under the conditions of simulated reduction in UV (UV-A and UV-B) radiation, to explore whether natural UV radiation reduction could stimulate tundra N_2_O and CH_4_ emissions. This is an important attempt to increase the Antarctic GHGs data sets to reasonably evaluate the potential effects of UV radiation reduction on tundra N_2_O and CH_4_ fluxes.

## Results

### UV radiation and environmental variables between experimental treatments

**Figure 2:**
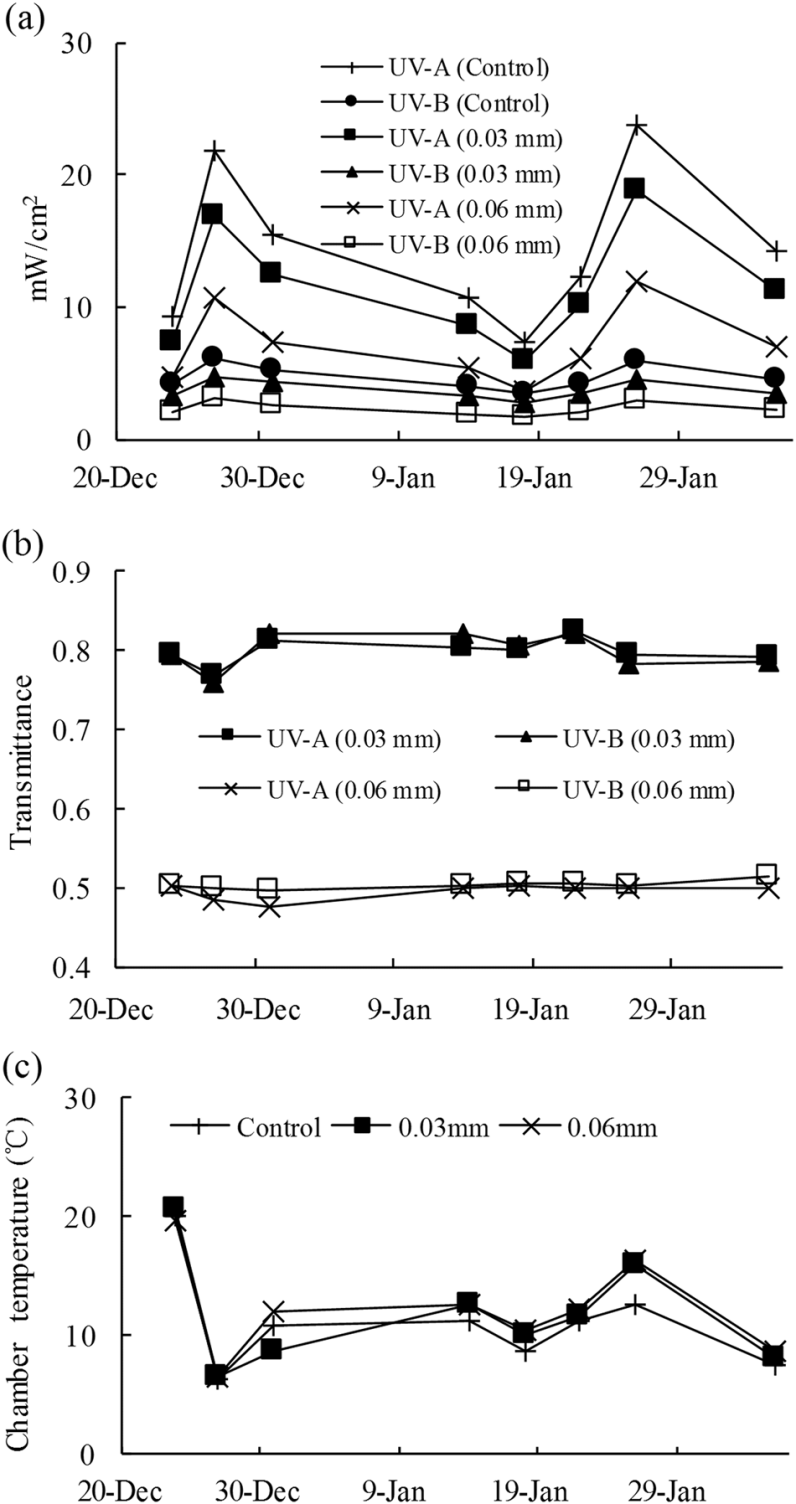
Variations in solar UV radiation intensity (**a**), transmittance (**b**) and chamber temperature (**c**) at the tundra sites with different thicknesses of UV radiation filter membrane.

In the summer of 2011/2012, UV radiation intensity showed similar temporal variation patterns between the control site and the sites covered by 0.03 mm and 0.06 mm filter membranes (Fig. 2a). The use of filter membrane between experimental treatments significantly decreased (analysis of variance (ANOVA) and least significant difference (LSD) test, P < 0.05) the amount of UV radiation penetrating into the chamber (Table 1). Compared with the control tundra site, the UV-A and UV-B through the sites with 0.03 mm and 0.06 mm filter membrane decreased by 20% and 50%, respectively (Fig. 2b). The highest mean UV-A and UV-B intensity occurred at the control site (14.4 ± 2.1 mW cm^−2^ and 4.7 ± 0.3 mW cm^−2^, respectively), followed by the site covered by 0.03 mm membrane (11.4 ± 1.6 mW cm^−2^ and 3.8 ± 0.3 mW cm^−2^, respectively) and the lowest at the site covered by 0.06 mm membrane (7.1 ± 1.0 mW cm^−2^ and 2.4 ± 0.2 mW cm^−2^, respectively). However, no significant differences (ANOVA and LSD test, P > 0.05) were found in terms of chamber temperatures (CTs) between the different treatment groups (Table 1) and the CTs showed similar temporal variation patterns at different tundra sites (Fig. 2c). Thus, the use of filter membranes between experimental treatments did not significantly alter chamber micrometeorological conditions, except for the UV intensity. Therefore, the filter membranes could be used to stimulate various UV intensities and explore the effects of UV radiation on tundra N_2_O and CH_4_ fluxes in maritime Antarctica. In addition, soil environmental properties, including pH, soil moisture, soil total organic carbon (TOC) and total nitrogen (TN) were similar to each other among the sites: AW1, AW2 and AW3 in the western tundra; AE1, AE2 and AE3 in the eastern tundra on Ardley Island; and GW1, GW2 and GW3 in the upland tundra on Fildes Peninsula. Detailed information about the climatic conditions and soil physiochemical properties is given in Supplementary Figures S1 and Tables S1 and S2.

**Table 1:** Comparisons of UV radiation intensity and chamber temperature from the tundra observation sites with different thickness of UV radiation filter membrane.

Variables	Control		0.03 mm		0.06 mm	
	Range	Mean ± SE	Range	Mean ± SE	Range	Mean ± SE
UV-A (mW/cm^2^)	7.5–23.8	14.4 ± 2.1	6.0–18.9	11.4 ± 1.6	3.8–11.9	7.1 ± 1.0
UV-B (mW/cm^2^)	3.4–6.2	4.7 ± 0.3	2.8–4.7	3.8 ± 0.3	1.7–3.1	2.4 ± 0.2
Chamber temperature (°C)	6.3–20.0	11.1 ± 1.5	6.6–20.7	11.8 ± 1.6	6.6–19.7	11.9 ± 1.5

Note: The use of filter membrane between experimental treatments significantly decreased (ANOVA and LSD test, P < 0.05) the UV (UV-A and UV-B) radiation into the chamber, no significant differences (ANOVA and LSD test, P > 0.05) were found in terms of chamber temperatures between different treatment groups.

### Tundra N_2_O fluxes under reduced UV radiation

**Figure 3:**
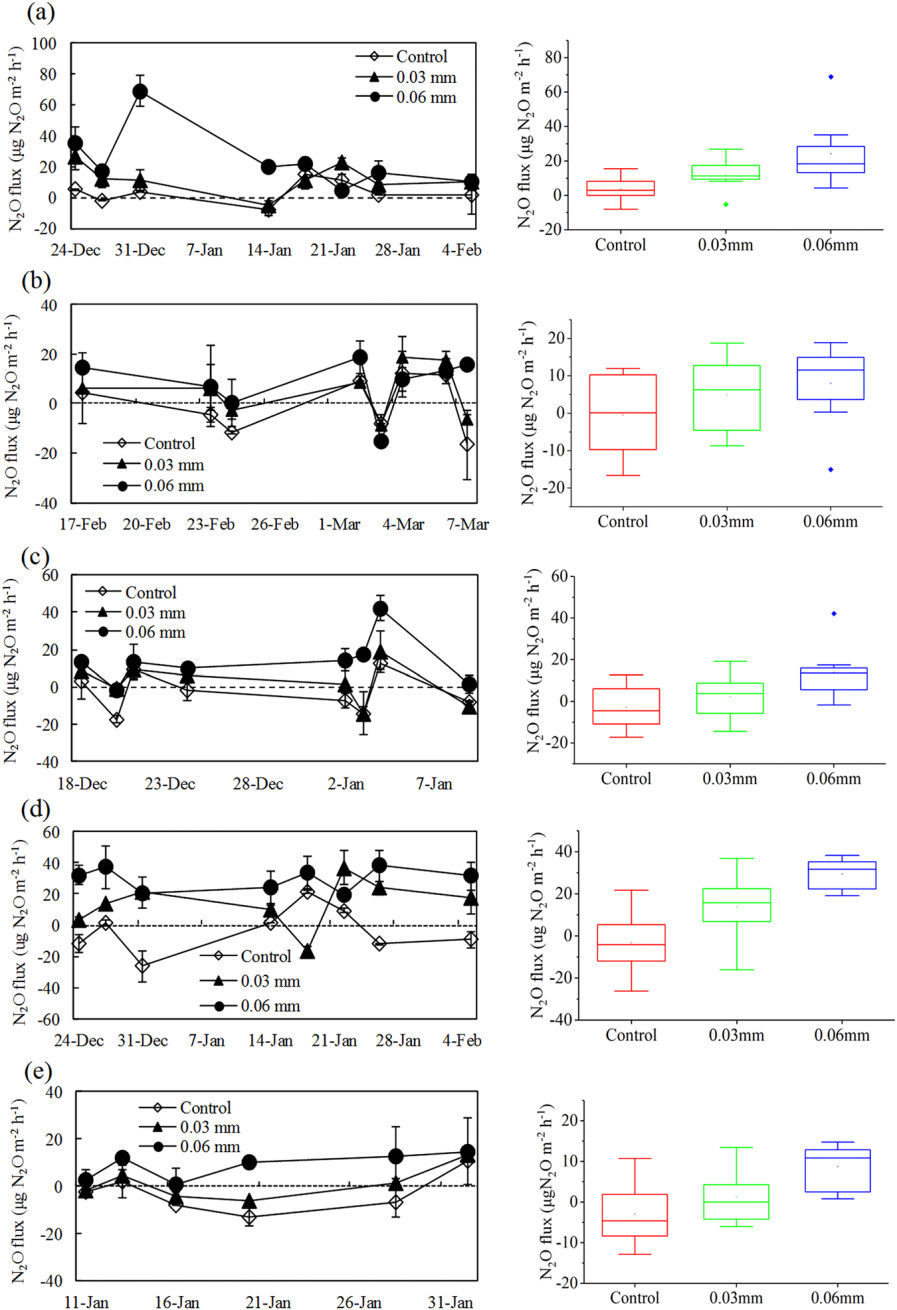
The N_2_O flux from the eastern, western and upland tundra sites with different experimental treatments under the reduction of UV radiation during the summers of 2011/2012, 2013/2014 and 2014/2015. Panels a–c show the western tundra N_2_O flux under the different UV radiation intensities in the summers of 2011/2012, 2013/2014 and 2014/2015, respectively; panel d shows the eastern tundra N_2_O flux under the different UV radiation intensities in 2011/2012 summer; and panel e shows the upland tundra N_2_O flux under the different UV radiation intensities in summer 2014/2015. The squares represent the mean fluxes and solid lines represent median values. Boxes enclose the interquartile range; whiskers show the full range. Analysis of variance (ANOVA) and the least significant difference (LSD) tests on the N_2_O emission rates from all three sites showed a significant difference (P < 0.05) between the sites with different UV-radiation treatments.

During the three observation periods, tundra N_2_O fluxes showed similar fluctuations between experimental treatments under reduced UV radiation (Fig. 3). In the western tundra marsh on Ardley Island, the highest mean N_2_O flux (24.2 ± 7.1 μg N_2_O m^−2^ h^−1^ in summer 2011/2012, 8.0 ± 3.6 μg N_2_O m^−2^ h^−1^ in summer 2013/2014 and 13.8 ± 4.7 μg N_2_O m^−2^ h^−1^ in summer 2014/2015) occurred at the site AW3 under 50% reduction in UV radiation, followed by AW2 (12.2 ± 3.4 μg N_2_O m^−2^ h^−1^ in summer 2011/2012, 4.8 ± 3.4 μg N_2_O m^−2^ h^−1^ in summer 2013/2014 and 2.3 ± 3.9 μg N_2_O m^−2^ h^−1^ in summer 2014/2015) under 20% reduction in UV radiation and the lowest was at the control site AW1 (mean fluxes were close to the detection limit) (Fig. 3a,b,c). Similarly, in the eastern tundra on Ardley Island substantial N_2_O emissions (mean 29.5 ± 2.6 μg N_2_O m^−2^ h^−1^) were observed at site AE3 under 50% reduction in UV radiation in summer 2011/2012, which was almost twice as high as that at site AE2 under 20% reduction in UV radiation (mean 13.8 ± 5.5 μg N_2_O m^−2^ h^−1^), whereas the control site AE1 was a weak N_2_O sink (mean −3.2 ± 5.2 μg N_2_O m^−2^ h^−1^) (Fig. 3d). For the upland tundra, site GW3 under 50% reduction in UV radiation showed the highest N_2_O emissions (mean 8.8 ± 3.6 μg N_2_O m^−2^ h^−1^) at all the sites in summer 2014/2015, whereas the control site GW1 was a weak N_2_O sink with a mean flux of −3.0 ± 1.2 μg N_2_O m^−2^ h^−1^ (Fig. 3e). Overall, the reduction in UV radiation significantly increased tundra N_2_O emissions in maritime Antarctica, although the N_2_O fluxes fluctuated markedly between the summers of 2011/2012, 2013/2014 and and 2014/2015.

**Figure 4:**
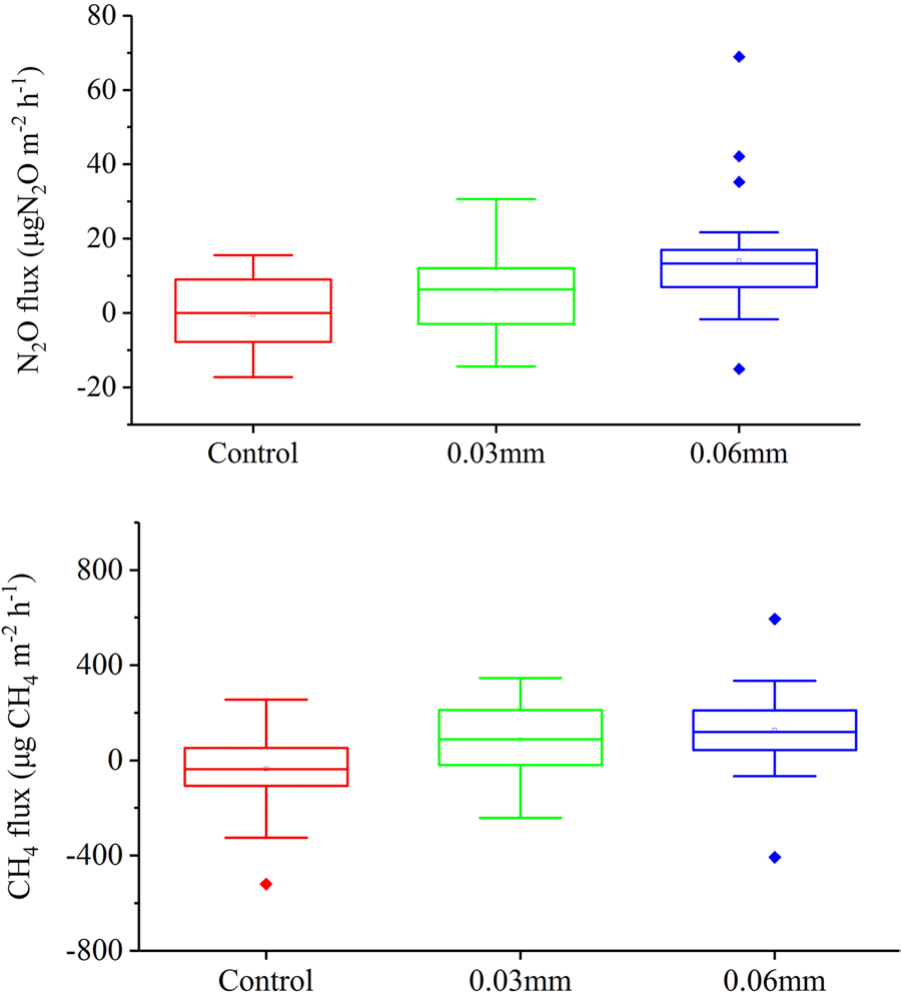
Comparisons of tundra N_2_O and CH_4_ fluxes under different UV radiation intensities in maritime Antarctica. Note: All the data from the sites AW1–AW3, AE1–AE3 and GW1–GW3 were analyzed for N_2_O and CH_4_ fluxes. For all the tundra sites, there were significant differences (ANOVA and LSD tests, P < 0.05) between the mean N_2_O, CH_4_ fluxes under the different UV radiation intensities.

ANOVA and LSD tests on the N_2_O emission rates from all three sites showed a significant difference (P < 0.05) among the sites with different UV-radiation treatments (Fig. 4). Relative to the controls, the 20% reduction in UV radiation increased the tundra N_2_O emissions by more than 5 μg N_2_O m^−2^ h^−1^, reaching as high as 14 μg N_2_O m^−2^ h^−1^. The 50% reduction in UV radiation increased tundra N_2_O emissions by more than 9 μg N_2_O m^−2^ h^−1^, reaching as high as 27 μg N_2_O m^−2^ h^−1^ during the observation periods (Table 2). Therefore, UV radiation intensity had an important effect on the N_2_O fluxes in maritime Antarctic tundra. Tundra N_2_O fluxes showed no significant correlations (Pearson correlation test, P > 0.05) with total organic carbon, soil moisture, total nitrogen, 0 cm soil temperature, 5 cm soil temperature, 10 cm soil temperature and NO_3_^−^-N and NH_4_^+^-N contents when the data at all the tundra sites were combined (Table S3), thus these environmental variables might not be the key factors affecting tundra N_2_O fluxes.

**Table 2:** Tundra N_2_O fluxes under different experimental treatments in the summers of 2011/2012, 2013/2014 and 2014/2015.

Observation period	Control		0.03 mm		0.06 mm		Difference	
	Range	Mean ± SE	Range	Mean ± SE	Range	Mean ± SE	CT_0.03_ (Control-0.03 mm)	CT_0.06_ (Control-0.06 mm)
2011/2012	−26.2–21.7	0.2 ± 3.9	−16.1–36.8	14.3 ± 4.8	4.4–68.9	26.8 ± 4.9	−14.1	−26.6
2013/2014	−16.6–11.9	−0.5 ± 3.1	−8.8–18.7	4.8 ± 3.4	−15.1–18.8	8.0 ± 3.6	−5.3	−8.5
2014/2015	−17.2–12.7	−2.9 ± 0.8	−14.4–19.1	1.9 ± 0.5	−1.7–42.1	11.7 ± 3.1	−4.8	−14.6
Comprehensive	−26.2–21.7	−1.1 ± 0.2	−16.1–36.8	7.2 ± 1.2	−15.1–68.9	17.3 ± 2.8	−8.3	−18.4

Note: The ultraviolet radiation through the control site was not affected, the solar UV radiation through the site with 0.03 mm polyester filter membrane decreased by 20% and through 0.06 mm decreased by 50%. Analysis of variance (ANOVA) and the Least Significant Difference (LSD) test on the N_2_O emission rates from all three sites showed a significant difference (P < 0.05) between the sites with different UV-radiation treatments.

### Tundra CH_4_ fluxes under reduced UV radiation

**Figure 5:**
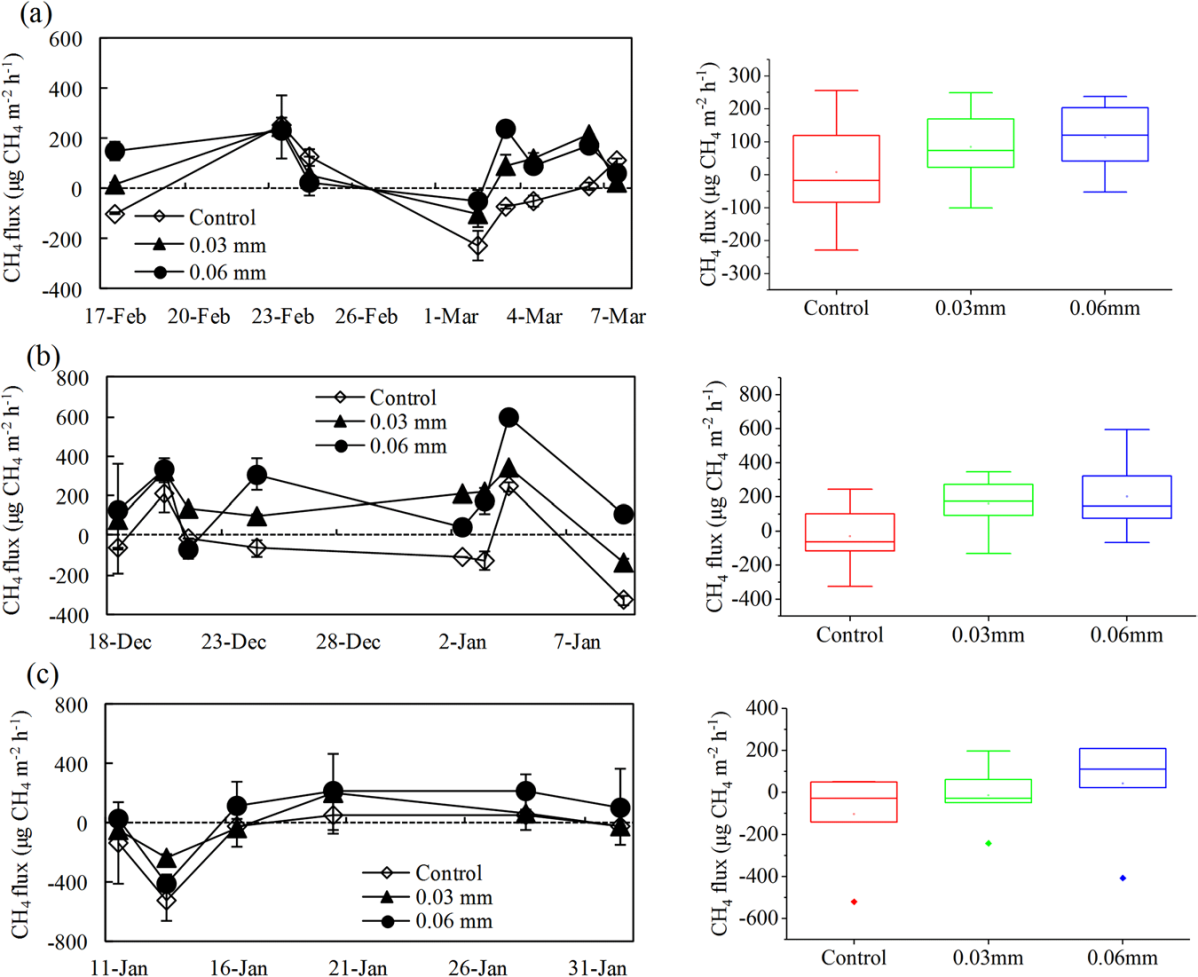
The CH_4_ flux from the western and upland tundra sites during the summers of 2013/2014 and 2014/2015. Panels a and b. shows the western tundra CH_4_ flux under the different UV radiation intensities in the summers of 2013/2014 and 2014/2015; panel c shows the upland tundra CH_4_ flux under the different UV radiation intensities in 2014/2015 summer. The squares represent the mean fluxes and solid lines represent median values. Boxes enclose the interquartile range; whiskers show the full range. ANOVA and the LSD test on the CH_4_ emission rates from all three sites showed a significant difference (P < 0.05) between the sites with different UV-radiation treatments.

During the summers of 2013/2014 and 2014/2015, the western tundra sites showed a large fluctuation, ranging from −324.9 to 594.4 μg CH_4_ m^−2^ h^−1^, with a mean of 89.5 ± 24.4 μg CH_4_ m^−2^ h^−1^ (Fig. 5a,b). Relatively strong CH_4_ uptake occurred at the control site AW1, with a mean flux of −11.4 ± 41.2 μg CH_4_ m^−2^ h^−1^. The flux at site AW2 under 20% reduction in UV radiation ranged between a weak sink and a weak source, with the mean of 122.4 ± 33.9 μg CH_4_ m^−2^ h^−1^. The CH_4_ flux at site AW3 under 50% reduction in UV radiation ranged between a weak sink (as low as −66.9 μg CH_4_ m^−2^ h^−1^) and a strong source (up to 594.4 μg CH_4_ m^−2^ h^−1^), with the greatest mean CH_4_ emission rate (157.7 ± 40.9 μg CH_4_ m^−2^ h^−1^) among all the sites. Similarly, the upland tundra acted as stronger CH_4_ sink at the control site GW1 (mean −102.4 ± 88.3 μg CH_4_ m^−2^ h^−1^ with the maximum uptake of 520.1 μg CH_4_ m^−2^ h^−1^) compared with site GW2 (mean −14.3 ± 58.9 μg CH_4_ m^−2^ h^−1^) under 20% reduction in UV radiation, whereas tundra site GW3 under 50% reduction in UV radiation showed weak CH_4_ emission (mean 42.5 ± 94.5 μg CH_4_ m^−2^ h^−1^) in summer 2014/2015 (Fig. 5c). Therefore, the reduction of UV radiation decreased tundra CH_4_ uptake rates over all three sites and could even convert the tundra from CH_4_ sinks into net sources in maritime Antarctica.

There were significant differences (ANOVA and LSD test, P < 0.05) between the mean CH_4_ fluxes under the different UV radiation intensities for all tundra sites (Fig. 4). Relative to the controls, the 20% reduction in UV intensity increased tundra CH_4_ emissions by more than 77 μg CH_4_ m^−2^ h^−1^, reaching as high as 109 μg CH_4_ m^−2^ h^−1^. The 50% reduction in UV intensity increased tundra CH_4_ emissions by more than 106 μg CH_4_ m^−2^ h^−1^, reaching as high as 150 μg CH_4_ m^−2^ h^−1^ during the observation periods (Table 3). Therefore, UV radiation intensity had an impact on tundra CH_4_ fluxes in maritime Antarctica. Except for 0 cm soil temperature, CH_4_ fluxes showed no significant correlations (Pearson correlation analysis, P > 0.05) with total organic carbon, soil moisture, total nitrogen, 5 cm soil temperature, 10 cm soil temperature and NO_3_^−^-N and NH_4_^+^-N contents (Table S3), indicating that these environmental variables might not be the key factors affecting tundra CH_4_ fluxes.

**Table 3:** Tundra CH_4_ fluxes under different experimental treatments in the summers of 2013/2014 and 2014/2015.

Observation period	Control		0.03 mm		0.06 mm		Difference	
	Range	Mean ± SE	Range	Mean ± SE	Range	Mean ± SE	CT_0.03_ (Control-0.03 mm)	CT_0.06_ (Control-0.06 mm)
2013/2014	−229.1–255.3	7.3 ± 50.4	−101.9–248.6	84.2 ± 37.4	−52.7–237.2	113.7 ± 33.9	−76.9	−106.4
2014/2015	−520.1–244.1	−61.1 ± 16.3	−242.1–345.9	85.6 ± 22.9	−407.4–594.4	133.4 ± 35.7	−109.3	−150.4
Comprehensive	−520.1–255.3	−36.2 ± 7.7	−242.1–345.9	85.1 ± 18.1	−407.4–594.4	126.2 ± 26.9	−121.3	−162.4

Note: The ultraviolet radiation through the control site was not affected, the solar UV radiation through the site with 0.03 mm polyester filter membrane decreased by 20% and through 0.06 mm decreased by 50%. Analysis of variance (ANOVA) and the Least Significant Difference (LSD) test on the CH_4_ emission rates from all three sites showed a significant difference (P < 0.05) between the sites with different UV-radiation treatments. The tundra CH_4_ was not observed in 2011/2012 summer.

## Discussion

In this study, no significant correlation (Pearson correlation analysis, P > 0.05) was found between tundra N_2_O fluxes and soil biogeochemical properties (Table S3). However, reduced UV radiation significantly (ANOVA and LSD test, P < 0.05) increased tundra N_2_O emissions in maritime Antarctica, confirming that the variability in UV radiation has an important effect on tundra N_2_O fluxes and a reduction in UV radiation might increase tundra vegetation N_2_O production. Some wetland plants can produce and release some N_2_O via the physiological reaction of plant tissues^33,34^. Generally nitrate reductase (NR), which is responsible for reducing nitrate into nitrite in some plants, plays a key role in the nitrogen metabolism pathway^26^. Furthermore, the reduction in UV radiation significantly stimulated the activities of NR and glutamine synthetase in plants^35,36^. In maritime Antarctica, tundra vegetation might also produce some N_2_O, which is probably related to the content of nitrate and the activity of NR. Indeed, exposure to enhanced UV radiation caused a decrease in the growth rate of *Deschampsia antarctica* and the activities of NR in maritime Antarctica^26^. Therefore, the reduction in UV radiation might increase NR activity, thereby stimulating nitrate reduction and N_2_O formation in tundra vegetation, which would lead to an increase in N_2_O emissions from tundra vegetation.

The increase in N_2_O emissions might also be caused by stimulation of tundra vegetation growth under reduced UV radiation. The response of tundra vegetation photosynthetic rates and vegetation-soil respiration rates to the change in light intensity was almost immediate in the static chambers^15,37^. Reduced UV radiation significantly increased photosynthesis, the leaf cross-section and the proportion of aerenchyma in most of wetland plants^34,36,38^. The growth of the two phanerogamic Antarctic plants, *Deschampsia antarctica* and *Colobanthus quitensis*, appeared to be affected by manipulated surface solar UV levels during the severe ozone depletion in field experiments^39^ and leaf growth of *Deschampsia antarctica* decreased with elevated UV-B^40^. Plant growth affected the available nitrogen, soluble organic carbon and O_2_ in the soil; and accelerated N_2_O production and release from the plant-soil system^14,38^. In addition, plants also serve as a conduit to transport the N_2_O produced in the soil to the atmosphere^14,36^. Therefore, the stimulation of tundra vegetation growth under reduced UV radiation might influence soil properties and further promote N_2_O emissions from the soil-vegetation system.

In addition, N_2_O is produced naturally through nitrification and denitrification by soil microorganisms^41^. Although UV radiation cannot penetrate into the soil below 5 mm, enhanced UV radiation may impose indirect effects on the dynamics of microbial communities, mainly via its direct influence on vegetation growth and physiological metabolism, which in turn reduces the absorption of available N and affects root secretion^42^. Many studies have shown that reduced UV radiation significantly increased total abundance and activities of bacteria, such as nitrifiers and denitrifiers, in the rhizosphere soil of wetland vegetation^26,34,43^. Therefore, reduced UV radiation might increase the activities of tundra soil microorganisms associated with the nitrogen cycle in maritime Antarctica.

Similarly, the lack of a significant correlation (Pearson correlation analysis, P > 0.05) between tundra CH_4_ fluxes and soil properties (Table S3) indicated that soil temperature, soil moisture and other soil properties had an insignificant effect on tundra CH_4_ fluxes. In this study, the reduction of UV intensity could significantly (ANOVA and LSD test, P < 0.05) increase tundra CH_4_ emission in maritime Antarctica, which was very similar to that observed at peatland sites in Finland^44^. Direct effects of UV radiation on CH_4_ producing or oxidizing bacteria were not likely because solar radiation penetrates only a few centimeters into the ground^45,46^. However, there are some indirect effects between UV radiation and CH_4_ emission, because the reduction of UV radiation induced changes in root exudates, which indirectly affect CH_4_ production in the soil^42,47^. Unlike higher plants, lichens and mosses in Antarctica lack a well-developed root system; therefore, most C/N organic material entering the extracellular pools in polar soils probably comes from root and microbial turnover^48,49^. Vegetation root exudates provide carbon and energy sources for the growth of methanogens, thus promoting CH_4_ production in the tundra^26,47^. Intense UV radiation might decrease the distribution of carbohydrates into the roots of vegetation in the Antarctic summer, which was thought to be the major reason why enhanced UV radiation inhibited CH_4_ emissions in wetlands^29,50^. UV radiation induced changes in the contents of soil root exudates and decreased UV radiation led to an increase of 15.8% in the rate of CH_4_ emissions from the wetlands^36^. Therefore, decreased UV radiation stimulated the secretion of root exudates, which might be an important mechanism underlying the effect of UV radiation on CH_4_ emissions from tundra wetland.

By contrast, in general, ground vegetation might exhibit morphological changes under different ultraviolet intensities^34,51^. Outdoor species may be sensitive to an increase in UV and decreased UV radiation significantly increased the leaf cross section and proportion of aerenchyma in most wetland plants^44,51^. In our study area, tundra vegetation, including short mosses and lichens, grow very close to the ground and some of them were even buried in the tundra soils^14^, therefore aerenchymatous tissue of tundra vegetation might have an important role in transporting CH_4_ from the soil to the atmosphere. In this experiment, the increased cross-sectional area of the plant aerenchyma caused by the reduction of UV radiation is one possible explanation for the stimulated transport of CH_4_ from the soil to the atmosphere. However, it remains unclear whether the stomatal functioning controls CH_4_ transport through the mosses or lichens. If the UV induces changes in the stomatal conductance of tundra plants, as shown in several studies with higher plants^44,52,53^, it could alter CH_4_ emission rates. Therefore, the reduction in UV radiation might stimulate CH_4_ emission by affecting tundra vegetation development.

In this study, atmospheric photochemical reactions in the chamber should also be considered. The UV-induced photolysis of N_2_O comprises approximately 90% of the global N_2_O sink^54^ and it is very likely that the enhanced N_2_O emissions under lower UV intensity were caused by reduced photolysis of N_2_O. In addition, an important atmospheric sink for CH_4_ is the reaction between OH and CH_4_^55^ and less OH might be generated when UV radiation is reduced in the chambers, thus the “apparent” CH_4_ flux from the tundra sites might also be enhanced when the chambers are covered by the thicker filter membranes. More research is needed to test these hypotheses in the future. In general, our results indicated that a reduction of natural UV radiation significantly (ANOVA and LSD test, P < 0.05) increased tundra N_2_O and CH_4_ emissions compared with the control under ambient UV levels (Tables 2 and 3). Solar UV radiation might have an important effect on N_2_O and CH_4_ budgets in the maritime Antarctic tundra. Although strong solar UV radiation still exists in maritime Antarctica, recovery of stratospheric ozone has occurred since the implementation of the Montreal Protocol in 1989 and the amount of solar UV radiation reaching the earth’s surface would be decreased^31,32^. The effects of UV radiation on tundra N_2_O and CH_4_ fluxes and their budgets, should be evaluated in the Arctic and Antarctic regions. The exclusion of its effects might underestimate N_2_O and CH_4_ budgets in the tundra ecosystem of Polar Regions. To assess the regional N_2_O and CH_4_ budget precisely, long-term measurements of GHG fluxes should be designed in the Antarctic or Arctic tundra ecosystems to show effects of UV radiation intensities on N_2_O and CH_4_ fluxes.

## Methods

### Study area and investigation sites

One research area was located on Ardley Island (62° 13′ S, 58° 56′W; an area of 2.0 × 1.5 km) (Fig. 1). This island is recognized by the Scientific Committee of Antarctic Research as an area of special scientific interest. The western region of this island is a costal lowland tundra marsh and the vegetation cover was around 95%^14^. The middle on this island is a non-level, hilly and relatively dry upland tundra, with vegetation coverage of 90–95%^14^. The middle upland and western lowland tundra are free of active penguin populations. The active penguin populations only concentrate in the east of this island^12^ and tundra patches have formed in the marginal zones of penguin nesting sites and are almost totally (90–95%) covered by mosses, algae and lichens in the east^15^.

Another research area was situated on Fildes Peninsula (61° 51′−62° 15′S, 57° 30′−59° 00′W; an area of 30 km^2^) in the southwestern area of King George Island (Fig. 1a,b). Communities of mosses and lichens represent the vegetation on this peninsula. An upland tundra was well-developed in the northwest of the Chinese Great Wall Station, at a distance of about 500 m from the station. The upland tundra was nearly dry, with an elevation of around 40 m a.s.l. The sampling ground was totally covered by mosses (*Bryum Pseudotriquetrum* and *Bryum muelenbeckii*) and lichens (*Usnea sp*.), with a depth of around 5–10 cm for the vegetation layer. Under the vegetation cover is an organic clay layer, with the depth of around 10–15 cm. A more detailed description about the study area was given by Zhu *et al*.^15^.

During the summers of 2011/2012, 2013/2014 and 2014/2015, three observation sites were set up in the western tundra marsh on Ardley Island, equipped with three chamber collars each. The chambers were covered by special polyester filter membranes (Mylar-D, 0.03-mm/0.06-mm thick; DuPont Co., Wilmington, DE, USA), which removed part of the UV-A and UV-B wavelengths and had no effect on other wavelengths of light^56^, to simulate the effect of natural UV-radiation reduction on tundra GHG fluxes: (1) the control site AW1 had transparent chambers; (2) site AW2 had transparent chambers covered by a 0.03-mm filter membrane; and (3) site AW3 had transparent chambers covered by a 0.06-mm filter membrane (Fig. 1c). In addition, during the summer of 2012/2013, three other observation sites were established in the eastern tundra of Ardley Island: (1) the control site AE1 had transparent chambers; (2) site AE2 had transparent chambers covered by a 0.03-mm filter membrane; and (3) site AE3 had transparent chambers covered by a 0.06-mm filter membrane (Fig. 1c). During summer 2014/2015, N_2_O and CH_4_ fluxes were also measured at three observation sites in the upland tundra on the Fildes Peninsula: (1) the control site GW1 had transparent chambers; (2) site GW2 had transparent chambers covered by a 0.03-mm filter membrane; (3) site GW3 had transparent chambers covered by a 0.06-mm filter membrane (Fig. 1b). There were no differences in the dominant vegetation species and phytomass among the three sites in each study area^15^. These observation sites were characteristic of the typical surface and vegetation within the tundra ecosystems in maritime Antarctica.

### UV radiation measurement

To test whether the UV radiation polyester filter membrane with different thicknesses could decrease solar ultraviolet radiation, we used an UV radiation instrument (Photoelectric Instrument Factory, Beijing Normal University, Beijing, China) with UV radiation sensors and data loggers (model UV-II) to measure the UV intensity. The sensors, which were manually mounted under the chambers with different thickness polyester filter membrane, collected UV data at 5-min intervals and the measured data displayed by the instrument was the radiant exposure (mW cm^−2^). The instrument was calibrated by the manufacturer and was used within the one-year interval of the validity for this calibration. The order of measurements was randomized to ensure that the measuring sequence did not bias the results and each site had three replicate measurements. During the period from Dec 24, 2011 to Feb 5, 2012, the UV radiation intensity was measured eight times at sites AW1, AW2 and AW3. These data indicated that the filter membrane significantly (ANOVA and LSD test, P < 0.05) decreased the UV radiation transmitted to the chamber (Fig. 2a). The UV radiation through site AW1 plots was not affected, the UV-A and UV-B decreased by 20% through the site AW2 plots and by 50% through the AW3 plots (Fig. 2b).

### *In situ* N_2_O and CH_4_ flux measurement

A static chamber technique was used to measure N_2_O and CH_4_ fluxes from the tundra sites^12,15^. Gas samples were taken from the clear plexiglass chambers (area: 0.25 m^2^, volume: 0.06 m^3^) placed on the PVC collars installed at the measurement sites. The collars were pushed 5 cm into the soil and air samples were taken within the headspace after 0, 10 and 20 min using a both ends needle. Gas samples were immediately transferred to 17.8 ml glass vials, which had been evacuated in advance^14,15^. More information on the *in situ* N_2_O and CH_4_ flux measurement is given in Supplementary Materials S1. During the summer of 2011/2012, N_2_O fluxes were measured at the sites (AW1, AW2, AW3 and AE1, AE2, AE3) from Dec 1, 2011 to Feb 21, 2012. During the summer of 2013/2014, N_2_O and CH_4_ fluxes were simultaneously measured at the western sites (AW1, AW2 and AW3) from Feb 14 to Mar 14, 2014. During the summer of 2014/2015, their fluxes were measured at the sites (AW1, AW2, AW3 and GW1, GW2, GW3) from Dec 1, 2014 to Feb 21, 2015.

### Analysis of N_2_O and CH_4_ concentrations and calculation of flux

The methods of analyzing N_2_O and CH_4_ concentrations and flux calculation were described in detail in our previous papers^12,15^. In brief, gas samples were analyzed using gas chromatography (GC-HP5890 II, USA; Shimadzu GC-14B, Japan; Shimadzu GC-12A, Japan) to measure N_2_O and CH_4_ concentrations. Their emission fluxes were calculated by fitting the experimental data to a linear least squares plot (N_2_O and CH_4_ concentrations *vs*. time). More information is given in Supplementary Materials S2.

### Measurements of environmental variables and soil properties

Soil temperatures (ST_0_, ST_5_ and ST_10_) were measured *in situ* using a ground thermometer inserted into the corresponding depth at the sampling sites. Meteorological data, e.g. air temperature (AT), daily sunlight time (ST), precipitation and total daily radiation (TDR) were acquired at the weather station of Great Wall Station. Soil samples were collected in the chamber plots after the fieldwork was completed in the summers of 2011/2012 and 2014/2015. The soils were sampled using a PVC tube (height: 15 cm; diameter: 6 cm), which was sealed and stored at 4 °C until analysis. Soil moisture was determined by oven drying at 105 °C to a constant weight. Each soil sample was homogenized manually and a subsample (fresh weight: 10 g) was extracted with 100 mL of 1 M KCl for 1 h and then filtered and analyzed for NH_4_^+^-N and NO_3_^−^-N, which were determined using a colorimetric method based on Berthelot’s reaction and ion chromatography^14,15^. The TOC content in the soils was determined by the chemical volumetric method^12^ and and TN was analyzed using automatic elemental analysis (Elementar Vario EL, Hanau, Germany). The pH was determined after a 1:3 (soil:solution) dilution of soil with distilled water^15^.

### Statistical analysis

The standard error (SE) was used to estimate the uncertainty of the mean of individual fluxes. All the data for N_2_O and CH_4_ fluxes were expressed as the mean ± SE. Differences in N_2_O fluxes or CH_4_ fluxes under different UV radiation intensities were examined using one-way repeated ANOVA and LSD tests at the P = 0.05 level. The relationships between soil parameters and N_2_O and CH_4_ fluxes were addressed using Pearson correlation analysis (P = 0.05 level). The contribution of the reduction in UV radiation to tundra N_2_O or CH_4_ fluxes was calculated as: CT_0.03_ = MF_0.03_-MF_con_ and CT_0.06_ = MF_0.06_-MF_con_. CT_0.03_ and CT_0.06_ indicate the contribution of the 20% and 50% reduction in UV radiation to tundra N_2_O or CH_4_ fluxes, respectively. MF_0.03_, MF_0.06_ and MF_con_ indicate the mean N_2_O or CH_4_ fluxes under the 20% and 50% reduction in UV radiation and under the control at the ambient UV level, respectively. All statistical analyses were performed using SPSS 20.0 (http://www.spss.com.cn/) and Microsoft Excel 2016 (https://products.office.com/zh-cn/excel) for Windows 10.

## Electronic supplementary material

Supplementary Material
