# Supplementary material for: Potential effects of ultraviolet radiation reduction on tundra nitrous oxide and methane fluxes in maritime Antarctica
Source: Sci Rep. 2018 Feb 27;8:3716. doi: 10.1038/s41598-018-21881-1 (PMC5829069; doi:10.1038/s41598-018-21881-1)
Supplement: Supplementary file 1 — Supplementary Material [file 41598_2018_21881_MOESM1_ESM.doc]

**Supplementary Material**

**Potential effects of ultraviolet radiation reduction on tundra nitrous oxide and methane fluxes in maritime Antarctica**

Tao Bao^1^, Renbin Zhu^1,*^, Pei Wang^1^, Wenjuan Ye^1^, Dawei Ma^1^& Hua Xu^2^

**S1. *In situ* N_2_O and CH_4_ flux measurement**

Open-bottomed clear plexiglass chambers (50×50×25 cm) were placed on the PVC collars installed at the measurement sites. These collars enclosed an area of about 0.25 m^2^ and were inserted into the soils to a depth of about 5 cm at each site. The use of flux collars allows the same spot to be measured repetitively, minimizes the site disturbance, and ensures that flux chambers are well sealed since the chambers fit into a water-filled notch in the collars. The average height of the chamber was 20 cm above the ground, which met the minimum required without influencing gas diffusion patterns that would prevail under normal atmospheric pressure.

During N_2_O and CH_4_ flux measurements, the chambers were inserted into the water-filled notch of the collars. Upon enclosure of the collars with cover chambers, headspace gas samples were collected at 0, 10 and 20 min intervals with a both ends needle connected to pre-evacuated glass vials (17.8 ml) stopped with butyl rubber septa. For each N_2_O and CH_4_ flux measurement, a total of three samples were withdrawn from each chamber at 10 min intervals after enclosure. Air temperature inside the chambers was simultaneously measured through the thermometer installed on the chambers. N_2_O and CH_4_ fluxes were determined between 9:00-11:00 (local time) at all the regular sites, resulting in three replicate measurements per site, and measuring order was varied to ensure that the measuring time did not bias the results.

**S2. Analysis of N_2_O and CH_4_ concentrations and calculation of flux**

In brief, N_2_O and CH_4_ concentrations were analyzed using gas chromatography equipped with a ^63^Ni electron capture detector, a flame ionization detector and a thermal conductivity detector, respectively (GC-HP5890 II, USA; Shimadzu GC-12A, Japan; Shimadzu GC-14B, Japan). Net N_2_O and CH_4_ fluxes were calculated using a linear least squares fit in the time series of concentrations with averaged chamber temperatures. The least squares regression lines “headspace N_2_O and CH_4_ concentrations versus time” were first visually inspected for abrupt changes in the direction of the flux, resulting from disturbances such as the chamber leakage or soil disturbances during sampling. The fluxes were usually omitted if the linear regression fitting had an r^2^ value smaller than 0.90. The detection limit of N_2_O and CH_4_ fluxes was ±1.5 µg N_2_O m^-2^ h^-1^ and ±5.0 µg CH_4_ m^-2^ h^-1^, respectively. For the fluxes, positive values indicate net emission to the atmosphere and negative values indicate net uptake from the atmosphere.

**S3. Climate conditions**

During the three austral summer periods of 2011-2015, air and ground temperatures showed large fluctuations (Fig. S1 and Table S1). The mean AT smoothly increased from December to January, and then declined until February. The mean air and ground temperatures in summer 2014/2015 (1.7 and 5.4 °C) were significantly higher (ANOVA and LSD test, P<0.01) than those in summer 2011/2012 (1.6 and 4.1 °C) and in summer 2013/2014 (1.3 and 4.3 °C). The daily minimum air and ground temperatures were often below 0 °C, while the daily maximum air and ground temperatures were generally above 5 °C. The total precipitation varied significantly (ANOVA and LSD test, P<0.01) between the summers of 2011/2012 (166 mm) and 2014/2015 (124 mm), and total sunlight time (ST) was 220 h and 153 h, respectively (Table S1). Overall, more precipitation occurred in summer 2011/2012 and summer 2014/2015 was relatively warmer and drier than summer 2011/2012.

**S4. Tundra soil characteristics**

Overall soil environmental variables including pH, soil moisture, soil TOC, TN were close to each other among the sites AW1, AW2 and AW3 in western tundra, AE1, AE2 and AE3 in eastern tundra on Ardley Island, and GW1, GW2 and GW3 in the upland tundra on Fildes Peninsula, respectively. However, soil physiochemical properties between tundra areas showed evident differences. Soil pH varied from 4.5 to 7.3 in the marsh and upland tundra, and the mean pH in marsh soils was slightly lower than that in upland tundra soils. The eastern tundra marsh soils at the AE1-AE3 adjacent to penguin colony were more acidic, and had lower C: N ratios than the western tundra marsh soils at the AW1-AW3 and the upland tundra soils at the GW1-GW3 far away from animal colonies (Table S2). Soil moisture between 81.0% and 88.9% was almost similar in tundra marsh soils, whereas Soil moisture (42.8-45.7%) in upland tundra soils were one half lower than those in the marsh soils. Particularly high soil inorganic nitrogen (NH_4_^+^-N and NO_3_^-^-N) contents occurred in the eastern tundra marsh soils with the means of 59.6 μg NH_4_^+^-N g^-1^ and 100.9 μg NO_3_^-^-N g^-1^. Soil TOC and TN contents ranged from 1.86 to 6.05% and from 0.23 to 0.58% in the western tundra marsh soils, whereas TOC (13.90-17.59%) and TN (2.01-2.36%) contents in eastern tundra soils were 2-3 times higher than those in the western marsh soils. Penguin activity and the deposition of their excreta significantly altered local soil physiochemical properties and contributed to generally low C: N ratios and the increase in soil TOC, TN, NH_4_^+^-N and NO_3_^-^-N contents.

**S5. Correlation between** **N_2_O and CH_4_ fluxes and other environmental variables**

As summarized in Table S3, the N_2_O and CH_4_ fluxes showed no significant correlations (P>0.05) with soil moisture (SM), total organic carbon (TOC), total nitrogen (TN), 0 cm soil temperature (ST_0_), 5 cm soil temperature (ST_5_), 10 cm soil temperature (ST_10_) and NH_4_^+^-N and NO_3_^−^-N contents when the data at all the tundra sites were combined. CH_4_ fluxes significantly correlated (r=−0.89, P<0.05) only with 0 cm soil temperature. Overall these environmental variables might not be the key factors affecting tundra N_2_O and CH_4_ fluxes in the maritime Antarctic tundra.

(a)

2011/2012 Summer

Ummer

2013/2014 Summer

Ummer

(b)

2014/2015 Summer

Ummer

(c)

**Figure S1.** Meteorological characteristics during the summertime in the study area. (a) 2011/2012 summer; (b) 2013/2014 summer; (c) 2014/2015 summer. Note: The data for total daily radiation (TDR) during 2013/2014 and 2014/2015 summer and the data for atmospheric moisture (AM) during 2011/2012 summer were not obtained from Chinese Great Wall Station. AT, P and ST indicated daily mean air temperature, precipitation, and sunlight time, respectively.

**Table S1**. Summary of Climatic Data Set in the Study Area During N_2_O and CH_4_ Flux Observation Period.

| Climatic factors | Summer 2011/2012 | Summer 2013/2014 | Summer 2014/2015 |
| --- | --- | --- | --- |
| Daily mean AT (°C ± SD) | 1.6±0.1 | 1.3±0.2 | 1.7±0.1 |
| Maximum AT (°C) | 5.9 | 6.5 | 7.5 |
| Minimum AT (°C) | -4.9 | -4.1 | -4.1 |
| Number of day at mean AT>0°C | 80 | 26 | 78 |
| Number of day at mean AT<0°C | 3 | 3 | 5 |
| Daily mean GT (°C ± SD) | 4.1±0.2 | 4.3±0.5 | 5.4±0.6 |
| Maximum GT (°C) | 13.0 | 16 | 17 |
| Minimum GT (°C) | -3.0 | -2.8 | -3 |
| Number of day at mean GT>0°C | 72 | 26 | 69 |
| Number of day at mean GT<0°C | 11 | 3 | 14 |
| Total precipitation (mm) | 166.3 | 27.2 | 124 |
| Total sunlight time (h) | 219.7 | 76.2 | 152.9 |

Note: AT and GT indicated daily air temperature and ground temperature, respectively.

**Table S2**. Soil physiochemical properties at the observation sites for tundra marsh on Ardley Island and upland tundra on Fildes Peninsula of maritime Antarctica (n = 3).

| Tundra sites | pH | SM  (%) | TOC  (%) | TN  (%) | NH_4_^+^-N (μg g^−1^) | NO_3_^−^-N (μg g^−1^) | C/N |
| --- | --- | --- | --- | --- | --- | --- | --- |
| Tundra marsh on Ardley Island (TM) | | | | | | | |
| AW1 | 6.5 | 85.8 | 2.26 | 0.27 | 9.84 | 15.61 | 8.4 |
| AW2 | 6.7 | 81.0 | 2.24 | 0.23 | 8.97 | 31.62 | 9.7 |
| AW3 | 6.7 | 84.8 | 1.86 | 0.24 | 7.47 | 13.26 | 7.8 |
| AE1 | 5.2 | 83.8 | 16.56 | 2.05 | 43.62 | 74.89 | 8.1 |
| AE2 | 4.9 | 82.3 | 13.90 | 2.08 | 31.93 | 44.67 | 6.7 |
| AE3 | 4.5 | 86.9 | 17.59 | 2.36 | 83.59 | 151.65 | 7.5 |
| Upland tundra on Fildes Peninsula (UT) | | | | | | | |
| GW1 | 7.3 | 45.7 | 1.86 | 0.08 | 1.26 | 0.53 | 9.4 |
| GW2 | 7.1 | 44.9 | 1.25 | 0.08 | 1.13 | 0.48 | 11.5 |
| GW3 | 6.8 | 42.8 | 1.08 | 0.12 | 1.17 | 0.44 | 8.5 |

Note: SM, TOC, TN, NH_4_^+^-N, NO_3_^−^-N, and C/N indicate soil moisture, total organic carbon, total nitrogen and the ratios of soil carbon and nitrogen, respectively.

**Table S3.** Pearson correlation coefficient between N_2_O and CH_4_ fluxes and soil physicochemical property during the observation period.

| Correlation | pH | SM  (%) | TOC  (%) | TN  (%) | NH_4_^+^-N (μg g^−1^) | NO_3_^−^-N (μg g^−1^) | ST_0_  (℃) | ST_5_  (℃) | ST_10_  (℃) |
| --- | --- | --- | --- | --- | --- | --- | --- | --- | --- |
| N_2_O Flux | -0.42 | -0.11 | -0.20 | 0.27 | 0.34 | -0.47 | 0.10 | 0.32 | 0.34 |
| CH_4_ Flux | 0.67 | -0.34 | -0.15 | -0.17 | -0.29 | -0.10 | -0.17 | -0.11 | 0.33 |

Note: **. Correlation is significant at the 0.01 level (1-tailed). *. Correlation is significant at the 0.05 level (1-tailed). SM, TOC and TN indicate soil moisture, total organic carbon and total nitrogen, respectively. ST_0_, ST_5_ an ST_10_ indicate 0 cm soil temperature, 5 cm soil temperature and 10 cm soil temperature, respectively.
